# Hydrogen Can Passivate Carbon Impurities in Mg-Doped GaN

**DOI:** 10.1186/s11671-020-3263-9

**Published:** 2020-02-10

**Authors:** Yuheng Zhang, Feng Liang, Degang Zhao, Desheng Jiang, Zongshun Liu, Jianjun Zhu, Jing Yang, Shuangtao Liu

**Affiliations:** 1 State Key Laboratory of Integrated Optoelectronics, Institute of Semiconductors, Chinese Academy of Sciences, Beijing, 100083 China; 2 Center of Materials Science and Optoelectronics Engineering, University of Chinese Academy of Sciences, Beijing, 100049 China

**Keywords:** Mg-doped GaN, Hydrogen and carbon impurities, BL band

## Abstract

The effect of unintentionally doped hydrogen on the properties of Mg-doped p-GaN samples grown via metal-organic chemical vapor deposition (MOCVD) is investigated through room temperature photoluminescence (PL) and Hall and secondary ion mass spectroscopy (SIMS) measurements. It is found that there is an interaction between the residual hydrogen and carbon impurities. An increase of the carbon doping concentration can increase resistivity of the p-GaN and weaken blue luminescence (BL) band intensity. However, when hydrogen incorporation increased with carbon doping concentration, the increase of resistivity caused by carbon impurity is weaken and the BL band intensity is enhanced. This suggests that the co-doped hydrogen not only passivate Mg_Ga_, but also can passivate carbon impurities in Mg-doped p-GaN.

## Introduction

GaN-based third-generation semiconductor materials and their alloys have attracted great attention [1] due to their broad applications including light-emitting diodes (LEDs) [2–4] and laser diodes (LDs) [5–7]. Although GaN-based photonic devices are widely commercialized, the relatively low hole concentration and high resistivity of p-type GaN still significantly limit the performance of such devices [8, 9]. Much research has been down to improve the p-type doping efficiency for III-nitrides [10, 11]. Hydrogen and carbon are two main residual impurities existing in the metal-organic chemical deposition (MOCVD)-grown Mg-doped GaN epilayers. It is well known that hydrogen impurities can passivate Mg in p-GaN [12]. On the other hand, carbon impurities can form many kinds of defects and increase the resistivity of Mg-doped p-GaN. Much research has done to decrease the hydrogen and carbon impurities. However, there are few investigations on the interaction of hydrogen and carbon impurities.

It is known that too much residual impurity of either hydrogen or carbon can cause high resistivity in as-grown Mg-doped GaN films. On account of the H-containing MOCVD growth environment, Mg is always passivated by hydrogen impurities, and a neutral Mg–H bond complex can be formed during film growth [13]. Fortunately, in a remarkable way, the group of Nakamura et al. [12] has firstly demonstrated that rapid thermal annealing in N_2_ ambient at a temperature > 700 °C can successfully dissociate Mg–H complexes and effectively remove the hydrogen atoms from Mg-doped GaN films.

For the past few years, with the research and development of long-wavelength multiple-quantum-well (MQW) devices, high-indium-content InGaN/GaN layers have been widely used as active layers. To avoid the segregation and structural degradation of MQW, relatively low growth temperature (< 1000 °C) and relatively low rapid thermal annealing temperature are required. However, the unintentionally doped carbon impurity concentration increases with decreasing growth temperature, which leads to a higher concentration of carbon impurity-related defects in GaN, existing in the forms of substitutional defects (C_N_), interstitial defects (C_i_), and complexes [14, 15]. These defects can act as either donors or deep-acceptor species and increase the resistivity of p-GaN significantly [16]. As a result, the low-temperature (LT)-grown Mg-doped p-type GaN films often show a higher resistivity than those grown at higher temperature. Contrary to our expectations, our research has found that p-GaN films with both high concentration of hydrogen and carbon impurities show relatively low resistivity.

In this work, three sets of Mg-doped GaN films with different concentrations of hydrogen and carbon residual impurities are investigated through secondary ion mass spectroscopy (SIMS), photoluminescence (PL), and Hall measurements. It is found that hydrogen can passivate the carbon impurities in the p-GaN, which points out a new direction to grow high-quality p-type GaN film.

## Experimental Methods

It still remains unknown on how to control the residual hydrogen concentration by setting MOCVD growth conditions. So, our samples are divided in different groups basing on SIMS results rather than growth conditions, similar Mg concentration in each group.

In this work, numbers of Mg-doped GaN films are grown on a 2-μm-thick unintentionally doped GaN layer template in a metal-organic chemical vapor deposition (MOCVD) system. Trimethylgallium (TMGa), ammonia (NH_3_), and bis-cyclopentadienyl-magnesium (Cp_2_Mg) are used as the precursors for Ga, N, and Mg, respectively. The growth temperature of all p-GaN samples is relatively low at 1020 °C. The Mg doping concentration is mainly adjusted by Cp_2_Mg flow rate. The residual carbon impurity concentration is adjusted mainly by NH_3_ flow rate during MOCVD—more NH_3_ corresponds to less carbon impurity [17]. The rapid thermal annealing is carried out in a nitrogen environment at a temperature of 800 °C for 3 min to de-passivate the Mg–H complexes.

Hall test is carried out to measure the resistivity of p-GaN samples. To make ohmic contact on p-type GaN, molten indium metal is pointed on a sample surface and acts as a metal electrode. To check the concentrations of magnesium, hydrogen, carbon, and oxygen impurities, [Mg], [C], [H], [O], secondary ion mass spectroscopy (SIMS) measurements of these p-GaN samples are taken. Seven samples are selected because of the suitable Mg concentration and divided in three groups, similar Mg concentration in each group, named as A1, A2, A3, B1, B2, and C1, C2.

Room temperature photoluminescence (PL) measurements of all samples are carried out by the 325-nm wavelength of a He–Cd laser at an excitation density of about 0.4 W/cm^2^. The luminescence intensity is normalized by the near-band-edge emission luminescence intensity (at around 3.44 eV)^1^ for the sake of analysis.

## Results and Discussion

The results of Hall test and SIMS measurement are exhibited in Table 1. Based on the SIMS results of Mg, C, and H concentration measurements, the seven samples are divided into three groups A, B, and C. Samples in each group has to be similar to Mg concentration, because Mg is the major acceptor in p-GaN and the conductivity of p-GaN is mostly caused by Mg. So, if we want to investigate the influence of H and C impurity on resistivity, we should keep Mg concentration invariability in each group. The joint influence of doping concentrations of these impurities on the sample property, mainly the p-type electrical resistivity, is analyzed. The doping concentration of magnesium in these samples is very high (in 10^19^~3 × 10^19^ cm^−3^) and has no remarkable difference for the samples in each group. The concentration of oxygen is low enough (10^16^ cm^−3^) and can be taken out of further consideration.

**Table 1:** Resistivity and Mg, C, H, and O concentrations for Mg-doped p-GaN samples

Sample	Resistivity (Ω cm)	Mg (cm^−3^)	C (cm^−3^)	H (cm^−3^)
A1	1.39	2.87E+19	1.17 E+17	1.70E+18
A2	4.95	3.14E+19	8.00E+17	8.70E+17
A3	47.7	2.39E+19	1.12E+19	4.11E+17
B1	1.49	1.10E+19	2.40E+16	4.60E+17
B2	2.35	1.08E+19	4.13E+17	2.81E+18
C1	1.95	1.60E+19	2.90E+17	1.50E+18
C2	2.95	1.40E+19	4.20E+17	4.59E+18

In group A, the rise of carbon impurities causes a tremendous resistivity increase of the p-GaN, while in group B, the increase of hydrogen along with carbon impurities is found to weaken this trend. And group C is employed to further investigate the influence on BL band.

**Fig. 1:**
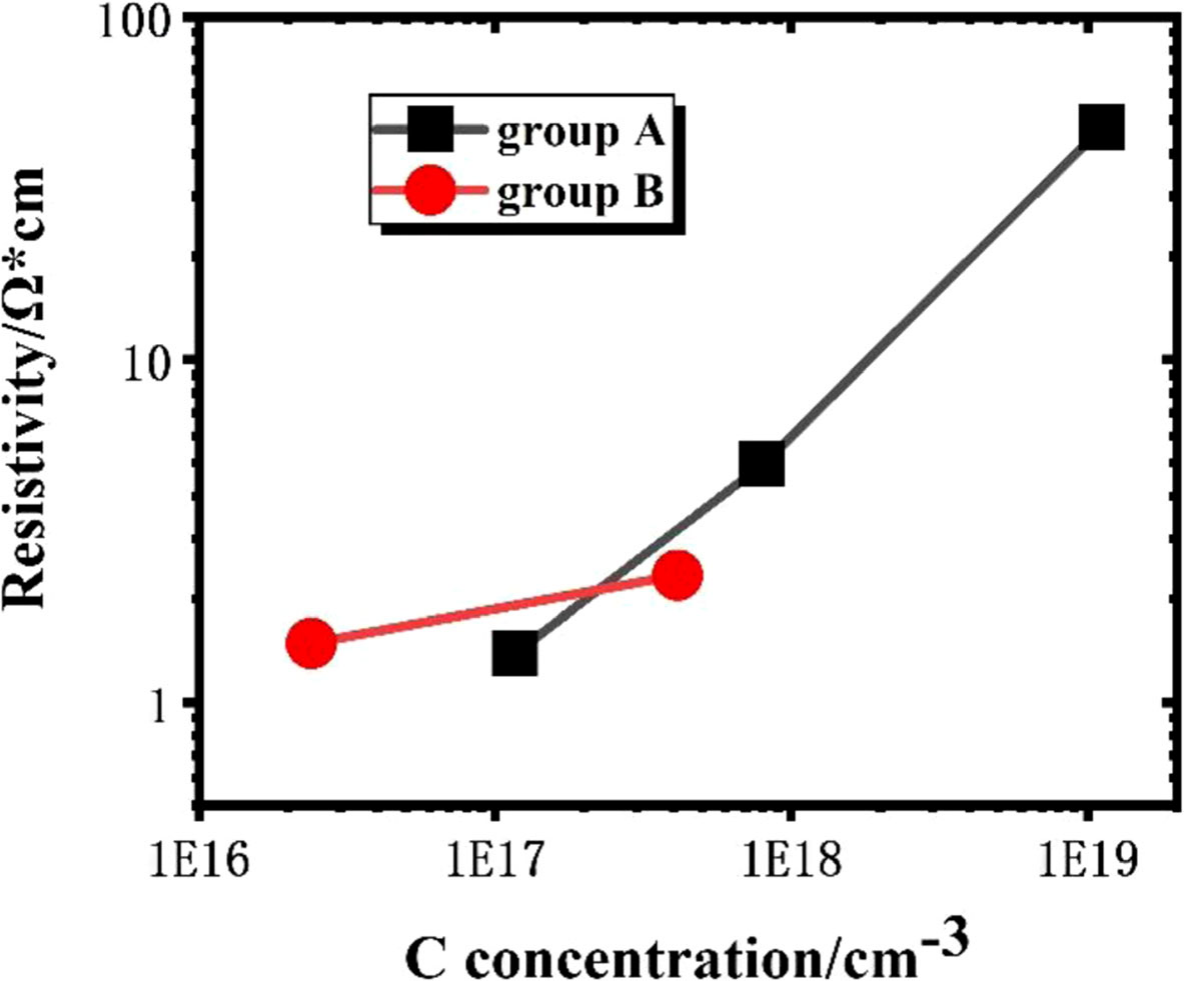
The resistivity of samples changes with the C concentration in groups A and B

It can be seen in Table 1 and Fig. 1 that for samples A1–A3, the concentration of carbon impurity increases dramatically, changing two orders of magnitude from 1.17 × 10^17^ to 1.12 × 10^19^ cm^− 3^, but the concentrations of magnesium, hydrogen, and oxygen change only little. From the previous research, we realized that though the doping concentration of magnesium is very high, actually the hole concentration is still two orders of magnitude lower than magnesium because of the low ionization rate and high possibility of self-compensation [18, 19]. In GaN, Mg_Ga_ has an acceptor ionization energy of 260 meV [20], an order of magnitude more than k_B_T (about 26 meV) in room temperature, and defects and impurities exist in GaN can compensate or passivate Mg_Ga_, so the hole concentration in Mg-doped GaN is about two orders of magnitude lower than magnesium. In addition, the residual carbon impurities can also cause negative effects to p-type GaN conductivity [16]. The resistivity of p-GaN samples in series A raised obviously with increasing carbon concentration (from 1.39 to ~ 47.7 Ω cm). Therefore, the differences between samples A1–A3 can be attributed to the difference of carbon impurities. As described in our previous study [16], carbon impurities may preferentially play the role of donor-type compensation centers in Mg-doped GaN films. The donors can compensate magnesium acceptors. Therefore, the resistivity of p-GaN increases with the rise of residual carbon impurities concentration.

On the other side, in series B, the concentrations of magnesium and oxygen change little in each group as shown in Table 1 and Fig. 1. The carbon concentration of sample B1 is much higher (about 20 times) than that of sample B2. However, the resistivity of sample B2 is quite close to and not much larger than that of sample B1. This trend is different from what we have observed for group A. Hence, it suggests that this different trend of resistivity variation in two groups may be attributed to the difference in the concentration of hydrogen impurity. For samples A1–A3, hydrogen impurity concentration decreases little, with a factor of ~ 1/3, while carbon impurity concentration increases nearly two orders of magnitude. On the contrary, for samples B1–B2, the concentration of hydrogen impurity increases along with the carbon impurity. Thus, the obtained result suggests that hydrogen incorporation may weaken the influence of carbon on the resistivity of Mg-doped p-GaN, producing a counteraction effect.

**Fig. 2:**
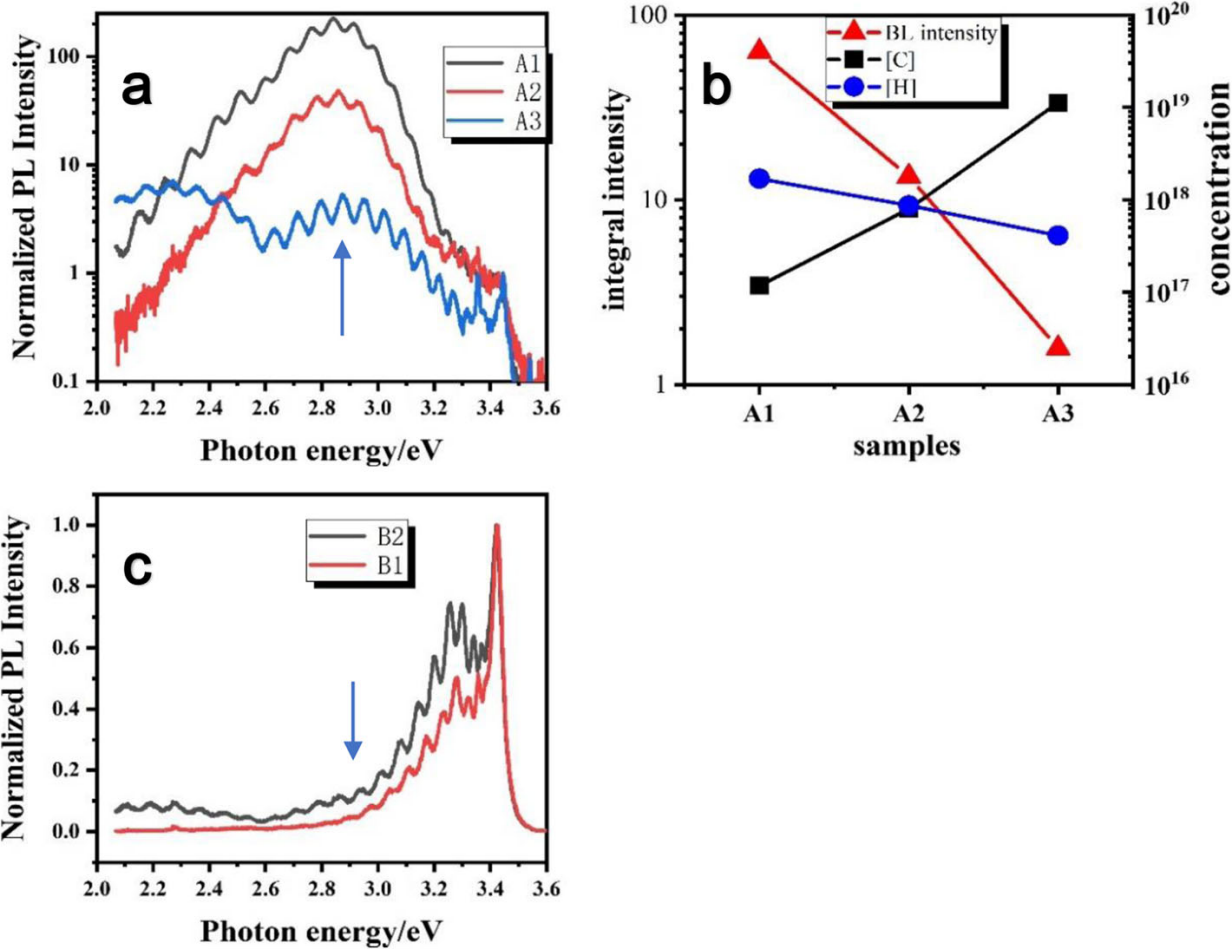
**a** The results of normalized PL intensity of samples A1~A3. **b** The integral PL (solid triangle) intensity and C (solid square) and H (solid circle) concentrations for samples A1~A3. **c** The results of normalized PL intensity of samples B1and B2

In order to further investigate how carbon impurity compensates magnesium acceptors and why hydrogen can weaken this process, the room temperature photoluminescence measurements were carried out. In Fig. 2a, as shown by the results of PL measurement of samples A1~A3, a luminescence peak at about 2.9 eV can be seen obviously. This blue luminescence (BL) band has been already studied for decades. It is known that the BL band in p-GaN PL spectra around 2.9 eV has a distinct donor–acceptor pair luminescence character. For the candidate of acceptor, isolated Mg substitute of Ga defect (Mg_Ga_) is the natural choice. And the most possible candidate for the deep donor in very heavily Mg-doped GaN is a nearest neighbor complex, which is an associate of Mg_Ga_ and nitrogen vacancy (V_N_), formed by self-compensation [21]. As the integral intensity of BL band decreases with higher doping of carbon impurities (Fig. 2b), we can assume that carbon impurities may decrease the number of relevant donor–acceptor pairs by compensating magnesium acceptors, because carbon impurities may preferentially play the role of donor-type compensation centers in Mg-doped GaN films [16]. The appearance of a strong 2.2-eV peak for sample A3 indicates that there is a larger number of carbon-related defects in sample A3 [15].

Meanwhile, regardless of a large increase of both carbon and hydrogen concentrations from B1 to B2 in sample group B, the PL spectra of these two samples are quite similar to each other. Actually, there is no obvious BL band in sample B1 and only a small BL peak in sample B2 (Fig. 2c), perhaps because of the relatively lower concentration of magnesium in series B samples (nearly 1 × 10^19^ cm^−3^) in comparison with group A samples. Therefore, the data of samples C1 and C2 are employed to check the interaction between hydrogen and carbon impurities further.

It is noted that the Mg and C concentrations in sample C1 are similar to those in sample C2, and the resistivity of the two samples is also similar to each other. But it is interesting to note that BL band changes obviously in the PL spectra of sample group C.

**Fig. 3:**
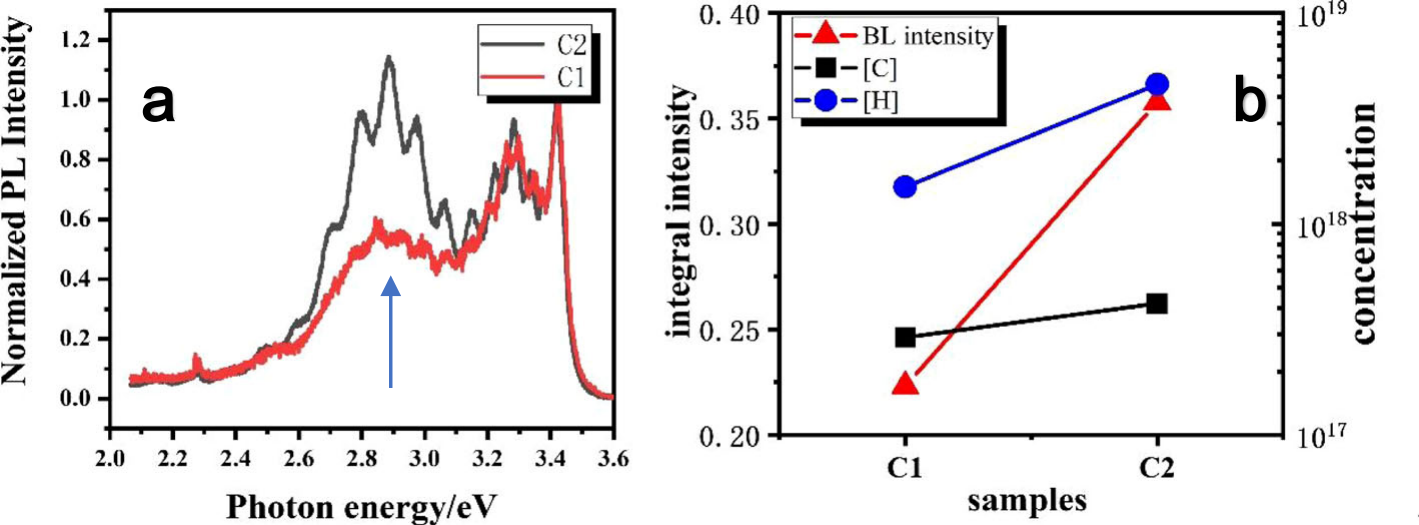
**a** The normalized PL intensity of samples C1 and C2. **b** Integral PL intensity and C and H concentration for samples C1 and C2

The H concentration in sample C2 is three times higher than that in sample C1. Figure 3a shows that the BL band intensity is quite different for samples C1 and C2. The intensity of BL band of C2 is much larger, which is attributed to the larger hydrogen concentration in this sample. In addition, the integral intensity of BL band increases clearly with the rise of the concentration of hydrogen, even though carbon impurity (can decrease BL band) concentration also increases a little at the same time (Fig. 3b). It implies that the reason for the increase of BL band is the increase of hydrogen impurities instead of carbon. It suggests that hydrogen and carbon may have an opposite effect on BL band of p-GaN. For hydrogen impurities, we assume that the most probable way to enhance the BL band is to form more relevant donor–acceptor pairs by forming C–H complexes with carbon impurity and passivating the carbon impurities in Mg-doped GaN. So, it is speculated that hydrogen can form complexes with carbon in the Mg-doped p-GaN sample, leading to a smaller concentration of donor-type compensation centers. In other words, hydrogen can passivate carbon and improve the conductivity of Mg-doped p-GaN. Further investigation is needed to figure out how to control hydrogen incorporation to preferentially passivate carbon impurity instead of Mg acceptors.

## Conclusion

In summary, the effects of carbon and hydrogen impurities on Mg-doped GaN films were investigated. It is found that carbon impurities may preferentially play the role of donor-type compensation centers and compensate Mg acceptor in Mg-doped GaN films. An increase of the carbon doping concentration can increase resistivity of the p-GaN and weaken blue luminescence (BL) band intensity. However, when hydrogen incorporation increased with carbon doping concentration, the increase of resistivity caused by carbon impurity is weaken and the BL band intensity is enhanced, which suggests that hydrogen not only can passivate Mg_Ga_ acceptors, but also may passivate carbon by forming C–H complex with carbon impurity.

## Data Availability

The datasets used and/or analyzed during the current study are available from the corresponding author on reasonable request.

## Abbreviations

GaN: Gallium nitride
InGaN: Indium gallium nitride
InN: Indium nitride
LD: Laser diode
LED: Light-emitting device
Mg_Ga_: Mg substitute of Ga defect
MOCVD: Metal-organic chemical deposition
MQW: Multiple quantum well
NH_3_: Ammonia
SIMS: Secondary ion mass spectroscopy
TMGa: Trimethylgallium
TMIn: Trimethylindium
V_N_: Nitrogen vacancy
